# Supplementary material for: Towards a One Health Food Safety Strategy for Palestine: A Mixed-Method Study
Source: Antibiotics (Basel). 2022 Oct 5;11(10):1359. doi: 10.3390/antibiotics11101359 (PMC9598066; doi:10.3390/antibiotics11101359)
Supplement: Supplementary file 1 [file antibiotics-11-01359-s001.zip › Supplementary Figure S1.pdf]

Supplementary Figure S1:

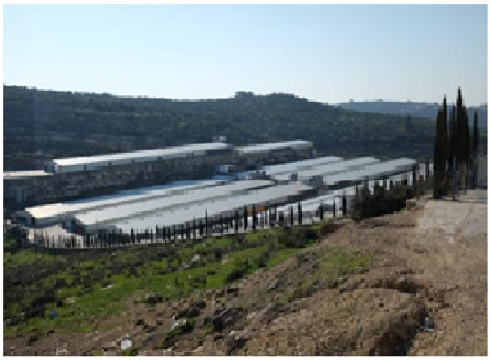

Supplementary Figure 1. a: Closed highly modernized buildings of a large-scale farm with biosecurity.

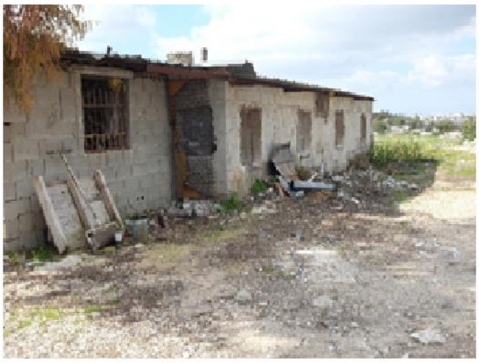

Supplementary figure 1. b: Simple small-scale farm with poor biosecurity.

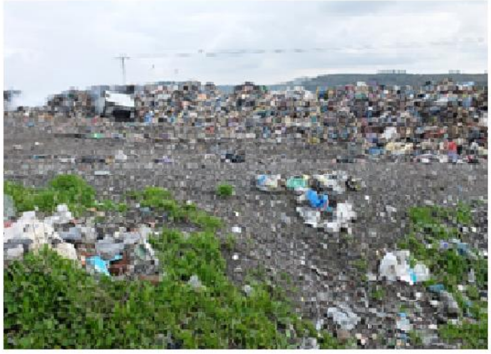

Supplementary Figure 1. c: Municipal dam, where among other things also dead animals are disposed of.

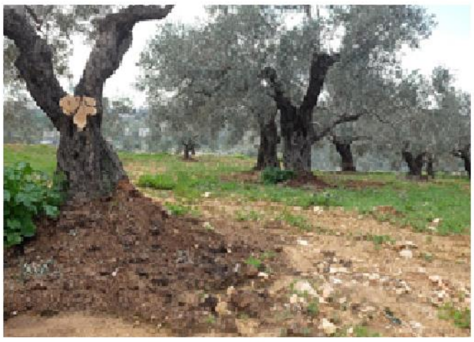

Supplementary Figure 1. d: Olive trees for which chicken manure is used as fertilizer.

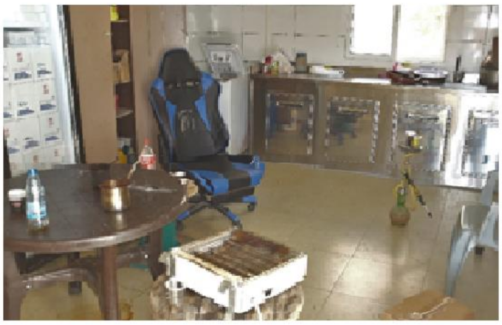

Supplementary Figure 1. e: Staffroom with kitchen and toilet in a separate building next to one of the large-scale farms visited

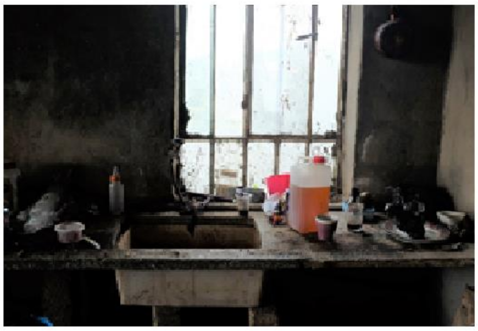

Supplementary Figure 1. f: Sink without soap or towel in a staffroom, which is separated from the small-scale farm only by a door
