# Supplementary material for: Towards a One Health Food Safety Strategy for Palestine: A Mixed-Method Study
Source: Antibiotics (Basel). 2022 Oct 5;11(10):1359. doi: 10.3390/antibiotics11101359 (PMC9598066; doi:10.3390/antibiotics11101359)
Supplement: Supplementary file 1 [file antibiotics-11-01359-s001.zip › Supplementary Figure S2.pdf]

Supplementary Figure S2:

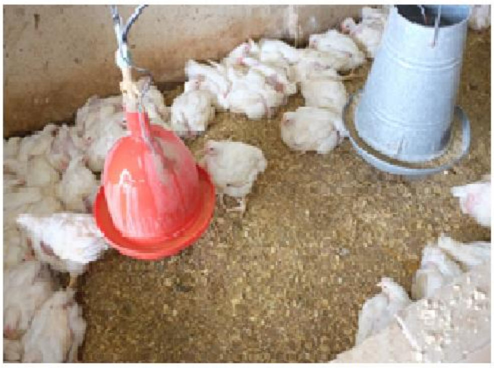

Supplementary Figure 2. a: Chickens kept in the interior of a traditional abattoir, ready to be selected for slaughter by consumers.

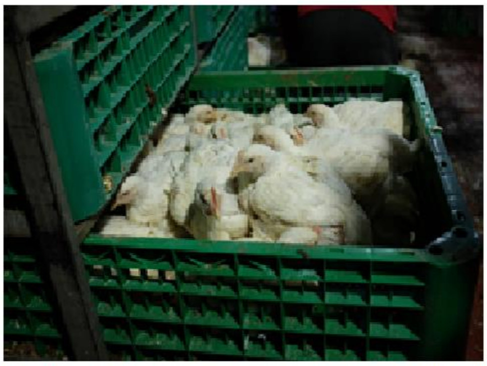

Supplementary figure 2. b: Chickens are delivered to public slaughterhouses in plastic boxes.

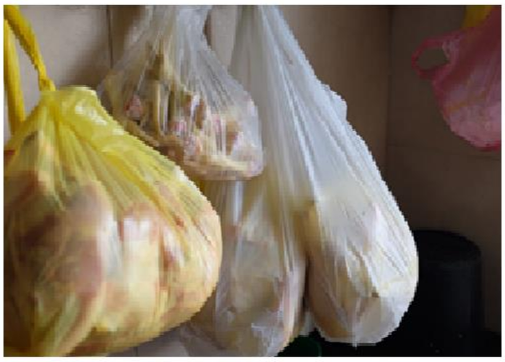

Supplementary Figure 2. c: Poultry meat or waste products such as chicken feet packed in plastic bags in a traditional abattoir.

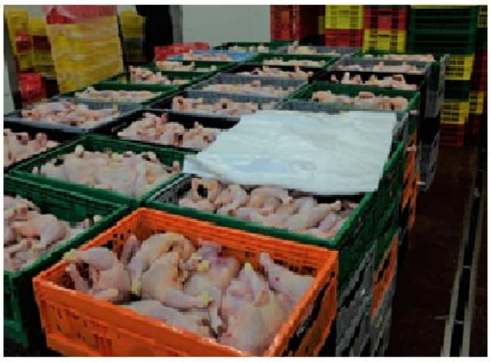

Supplementary Figure 2. d: Fresh assorted poultry packed in plastic boxes in a public slaughterhouse.
