# Supplementary material for: Towards a One Health Food Safety Strategy for Palestine: A Mixed-Method Study
Source: Antibiotics (Basel). 2022 Oct 5;11(10):1359. doi: 10.3390/antibiotics11101359 (PMC9598066; doi:10.3390/antibiotics11101359)
Supplement: Supplementary file 1 [file antibiotics-11-01359-s001.zip › Supplementary Figure S3.pdf]

Supplementary Figure S3:

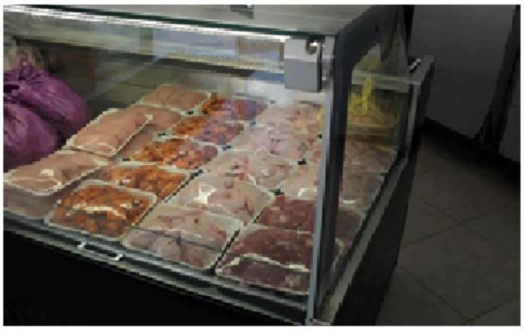

Supplementary Figure 3. a: Poultry is sold assorted, packaged, and refrigerated in meat stores.

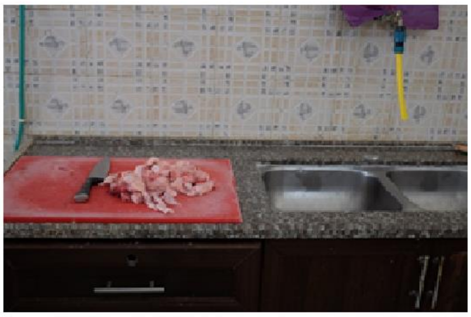

Supplementary Figure 3. b: Poultry remains lying next to the sink, which the meat seller tried to hide during our visit
