# Supplementary material for: Towards a One Health Food Safety Strategy for Palestine: A Mixed-Method Study
Source: Antibiotics (Basel). 2022 Oct 5;11(10):1359. doi: 10.3390/antibiotics11101359 (PMC9598066; doi:10.3390/antibiotics11101359)
Supplement: Supplementary file 1 [file antibiotics-11-01359-s001.zip › Supplementary Table S1.pdf]

Supplementary Table S1: Participants in the multi-stakeholder discussion groups who represented the relevant sectors.

Date	Discussion group	Relevant sectors	No. of participants
July 7, 2021	First discussion group	Ministry of Health	3
		Ministry of Agriculture/ Veterinary Services	2
		Ministry of Local Governance	2
		Ministry of National Economy	1
		Academic sector	3
July 8, 2021	Second discussion group	Ministry of Health/ Environmental Health Department	2
		Palestinian Veterinarians Syndicate	1
		Private veterinarians specialize in poultry disease	2
		Poultry producers	3
February 9, 2022	Third discussion group	Ministry of Health/ Environmental Health Department	2
		Ministry of Agriculture / Veterinary Services	2
		Ministry of Agriculture / Monitoring and Control Department	2
		Ministry of Local Governance	1
		Non-Government Organizations	2
		Academic sector	3
		Industrial sector	3
