# Supplementary material for: Towards a One Health Food Safety Strategy for Palestine: A Mixed-Method Study
Source: Antibiotics (Basel). 2022 Oct 5;11(10):1359. doi: 10.3390/antibiotics11101359 (PMC9598066; doi:10.3390/antibiotics11101359)
Supplement: Supplementary file 1 [file antibiotics-11-01359-s001.zip › Supplementary Table S2.pdf]

**Supplementary Table S2.**

**Semi-structured observational tool**

| | | |
|---|---|---|
| 1 | Name of the interviewer | |
| 2 | Date | \|___\|___\|/\|__\|___\|_2022 |
| 3 | Name of the settlement | |
| 4 | Farm/abattoir no. | |

To be completed by the interviewer before starting the interview

**General** observations

| | | |
|---|---|---|
| | Allowance to take photos (without showing the workers' faces)? | Yes (photos allowed)<br>No (no photos allowed) |
| 5 | Would you please show me where you retain water? | 1. Observed<br>2. Not observed |
| 6 | Would you please show me where you wash your hands? | 1. Observed<br>2. Not observed |
| 7 | Observe the availability of water and other products for cleaning hands in the area. | 1. Water, soap, and towel<br>2. Water and soap<br>3. Water and towel<br>4. Water alone<br>5. No water available<br>6. Other (specify) |
| 8 | Would you please show me your toilet? | 1. Observed<br>2. Not observed |
| 9 | Is the toilet inside or outside of the farm? | 1. Inside<br>2. Outside |
| 10 | Observe the cleanness of the toilet. | 1. Clean: no feces or urine outside the pit<br>2. Not clean: feces or urine outside the pit |

For **farms** only

| | | |
|---|---|---|
| 11 | Would you please show me your kitchen and where you sleep? | 1. Observed<br>2. Not observed |
| 12 | Observe the cleanness of the kitchen and bedroom. | 1. Open to the toilet<br>2. Open to poultry herd<br>3. Open to feed store<br>4. Separated |
| 13 | Would you please show me how you get rid of the dead poultry | 1. Observed<br>2. Not observed |

| | | |
|---|---|---|
| | and dung? | |
| 14 | Would you please show me your herd (to observe the visitor rules in terms of hygiene practices)? | 1. Observed<br>2. Not observed |

For **abattoirs** only

| | | |
|---|---|---|
| 15 | Would you please show me how you get rid of the slaughter waste (feathers, internal viscera, legs, poultry heads, etc.)? | 1. Observed<br>2. Not observed |
| 16 | Would you please show me the workers' kitchen? | 1. Observed<br>2. Not observed |
| 17 | Would you please show me the changing room for workers? | 1. Observed<br>2. Not observed |
| 18 | Would you please show me the different areas of your abattoir (from the hanging area to the last stage before marketing)? | 1. Observed<br>2. Not observed |
| 19 | Would you please show me the workers during the slaughtering process (from slaughtering to chilling)? | 1. Observed<br>2. Not observed |
