# Supplementary material for: Towards a One Health Food Safety Strategy for Palestine: A Mixed-Method Study
Source: Antibiotics (Basel). 2022 Oct 5;11(10):1359. doi: 10.3390/antibiotics11101359 (PMC9598066; doi:10.3390/antibiotics11101359)
Supplement: Supplementary file 1 [file antibiotics-11-01359-s001.zip › Supplementary Table S3.pdf]

Supplementary Table S3.

Codebook

Theme 1: The current system for monitoring food production

Category	Code	*Code description*
Epidemiological investigations	Pathogen testing and case detection	*Animals, meat, humans, and the environment can all be tested for pathogens. The results need to be properly communicated for case detection.*
	Follow-up of confirmed cases (test & slaughter)	*Once a case is detected, action must follow.*
Surveillance tools	Periodic examinations (from farm to fork)	*Regularly conducted examinations (by eye or laboratory testing) without suspicion at different stages along the food production chain: farms, transport, slaughter, markets, and food products.*
	Licensing of farms	*Palestinian law requires licensing of all farms, but many farms are unlicensed (random farms).*
	Veterinary health certificates	*Veterinary health certificates must be issued by a recognized veterinarian before slaughter.*

Theme 2: Regulatory authorities with responsibilities relevant to food safety

Category	Code	*Code description*
Organization of regulatory authorities	Responsibilities of the regulatory authorities	*Clear role distribution between regulatory authorities.*
	Cooperation between regulatory authorities	*Regulatory authorities working together on a particular issue.*
	Overlaps/conflicts between regulatory authorities	*Lack of clear role distribution between regulatory authorities.*
Initiatives and strategies	Toward a national food safety surveillance	*Advances toward a national food safety surveillance plan and its implementation.*
	Toward a national AMR surveillance	*Advances toward a national AMR surveillance plan and its implementation.*

Theme 3: Public health

Category	Code	*Code description*
Drugs in human and veterinary medicine	Human medicine: drug prescription and use	*Standards, regulations, and usage of drugs, especially antimicrobials, in human medicine.*
	Veterinary medicine: drug prescription and use	*Standards, regulations, and usage of drugs, especially antimicrobials, in veterinary medicine with a focus on adherence to the safety period/withdrawal period.*
	Drug residues and AMR	*Drug residues in meat or the environment and the associated growing AMR.*
Worker health and safety	Health insurance	*Health insurance system for workers involved in the food production chain (farmers, butchers, traders, veterinarians, etc.).*
	Financial insurance and economic incentives	*Financial insurance system for workers involved in the food production chain (farmers, butchers, traders, veterinarians, etc.) In the context of their economic incentives.*
	Legal protection	*Legal protection of workers involved in the food production chain (farmers, butchers, traders, veterinarians, etc.).*
Consumer health and safety	Hygiene practices and contamination (along the food production chain)	*Hygiene practices and associated contamination from farm to fork (farms, transport, slaughter, markets) threatening consumer health and safety.*
	Salmonella as an indicator of inadequate hygiene practices (in poultry production)	*Salmonella contamination in poultry production as a prime example of consumer health and safety hazards.*

	Prevention measures for food safety	*Measures to prevent foodborne disease may include vaccinations, pasteurization, raising consumers' awareness, the guidance of farmers, etc.*

Theme 4: Available infrastructure and capacity building

**Category**	**Code**	***Code description***
Expanding Infrastructure	Laboratories	*Requirements for laboratories in the context of food safety monitoring.*
	Slaughterhouses	*Requirements for slaughterhouses in the context of food safety monitoring.*
Expanding human resources	Veterinary services	*Expansion of veterinary services in terms of veterinarians' education, training, availability, etc.*
	Food and health inspectors	*Expansion of services provided by food and health inspectors in terms of inspectors' education, training, availability, etc.*

Theme 5: Political and legal context

**Category**	**Code**	***Code description***
Palestinian policy	Gap between the private and government sector	*Gaps between the private and government sector can manifest themselves through lacking guidance for farmers etc.*
	Regional differences	*Differences between Palestinian regions A, B, and C as well as differences between provinces or cities.*
	Covid-19 consequences	*Direct and indirect consequences of the Covid-19 pandemic.*
Palestinian legislation	Food law	*The role and importance of the Palestinian food law in the context of food safety.*
	Border control and smuggling	*Imports and exports of animals and food products to and from Palestine.*
